# Slow-Channel Congenital Myasthenic Syndrome Due to the Novel Variant c.1396G_A in CHRNA1 That Responds Favorably to 3,4-Diaminopyridine: A Case Report

**DOI:** 10.7759/cureus.73601

**Published:** 2024-11-13

**Authors:** Josef Finsterer

**Affiliations:** 1 Neurology, Neurology and Neurophysiology Center, Vienna, AUT

**Keywords:** 3/4-diaminopyridine, chrna1, congenital myasthenic syndrome, postsynaptic, repetitive nerve stimulation

## Abstract

Mutations in *CHRNA1* are responsible for postsynaptic congenital myasthenic syndromes (CMS) and occur either as slow-channel syndrome or fast-channel syndrome. Slow-channel CMS due to *CHRNA1* variants responds favorably to pyridostigmine. A patient with slow-channel CMS due to a new *CHRNA1* variant that responds favorably to 3,4-diaminopyridine (3,4-DAP) has not yet been reported.

The patient is a 36-year-old woman who was diagnosed with non-specific CMS at the age of one year when she presented clinically with signs of somnolence, weakness, and facial dysmorphism. She later also developed limb weakness, with the upper limbs being more severely affected. Heat, low humidity, late menstruation, high fever, and stress aggravated the muscle weakness. Only at the age of 17 was pyridostigmine started, which partially improved the muscle weakness. The diagnosis was genetically confirmed when the new, homozygous variant NM_001039523:c.1396G>A in *CHRNA1* p.(Gly466Arg) was detected at the age of 30. Since then, 3,4-DAP has been administered, which further improved the muscle weakness.

In summary, *CHRNA1*-associated slow-channel CMS may respond favorably not only to pyridostigmine but also to the additional administration of 3,4-DAP. Patients with *CHRNA1*-associated CMS can live for years without treatment, especially in early life. CMS should be diagnosed without delay to avoid putting people at risk of receiving medication that could potentially worsen their phenotype.

## Introduction

Congenital myasthenic syndromes (CMS) are a genetically and phenotypically heterogeneous group of hereditary transmission disorders that are usually associated with a multisystem disease that predominantly affects skeletal muscle [1]. CMS are pathophysiologically classified into postsynaptic, synaptic, presynaptic, and glycosylation-associated diseases [2]. Functionally, CMS are categorized as slow-channel, fast-channel, or deficiency syndromes [1]. Currently, 35 mutated genes are responsible for CMS phenotypes [3]. The most frequently mutated genes include *CHAT*, *COLQ*, *RAPSN*, *CHRNE*, *DOK7*, and *GFPT1* [2]. Phenotypically, mutations in CMS-related genes manifest as abnormal fatigability or permanent or fluctuating weakness of extraocular, facial, bulbar, axial, respiratory, or limb muscles, hypotonia, or developmental delay. Cognitive impairment, dysmorphism, neuropathy, or epilepsy may be rare manifestations of CMS [2]. Low- or high-frequency repetitive nerve stimulation may show an abnormal increase or decrease, and single-fiber electromyography may show increased jitter or blocks [2]. Most CMS respond favorably to acetylcholine esterase inhibitors, 3,4-diamino-pyridine (3,4-DAP), salbutamol, albuterol, ephedrine, fluoxetine, or atracurium [2].

One of the rare mutated genes that cause CMS is *CHRNA1* [4]. Mutations in *CHRNA1* are responsible for postsynaptic CMS and occur either as slow-channel syndrome [5] or fast-channel syndrome [6]. The phenotype of *CHRNA1* variants is similar to that of CMS [2], but individual patients may also exhibit arthrogryposis [4]. Slow-channel CMS due to *CHRNA1* mutations usually responds well to acetylcholinesterase inhibitors such as pyridostigmine [7]. Salbutamol or ephedrine are recommended as second-line therapies and 3,4-diaminopyridine (3,4-DAP) as third-line therapy [7]. There are also other studies reporting 3,4-DAP as effective for *CHRNA1*-associated CMS [3]. To our knowledge, a patient with slow-channel CMS due to the new variant c.1396G>A in *CHRNA1* who responds favorably to 3,4-DAP has not yet been reported.

## Case presentation

The patient is a 36-year-old woman who was diagnosed with non-specific CMS at the age of one year. The clinical manifestations of the disease began eight hours after birth when the facial features showed the first signs of drowsiness. As a baby and child, she did not have an open mouth. The muscle weakness affected all muscles but especially the hand and forearm muscles. In particular, taking a glass out of a cupboard or washing her hair could become difficult. Sometimes, the weakness also affected the lower legs but much less often than the forearms. Movement improved the muscle weakness. She could walk for four hours on a flat surface and felt better afterward. Heat, low humidity, late period, high fever, and stress exacerbated the muscle weakness. In addition to CMS, polycystic ovary syndrome (PCOS) was diagnosed in the medical history.

She received no treatment for CMS until the age of 17 when she was started on pyridostigmine at a dose of 120 mg/day. After about six months, the dose was increased to the current dose. The diagnosis was genetically confirmed at the age of 30 when the new, homozygous variant NM_001039523:c.1396G>A in *CHRNA1* p.(Gly466Arg) was discovered at the age of 30. After in silico evaluation, the variant was classified as probably deleterious (PolyPhen), C0 (Align GVGD), deleterious (SIFT), and disease-causing (mutation Taster). At the age of 30, she was administered 3.4-DAP (initially 10 mg/day), which significantly improved her muscle weakness. Since the age of 35, she also noticed weakness in the right index finger without any pathology of the median or ulnar nerve. The family history was negative for CMS, but none of the first-degree relatives were tested for the *CHRNA1* variant of the index patient. Her father's parents were first cousins.

**Figure 1:**
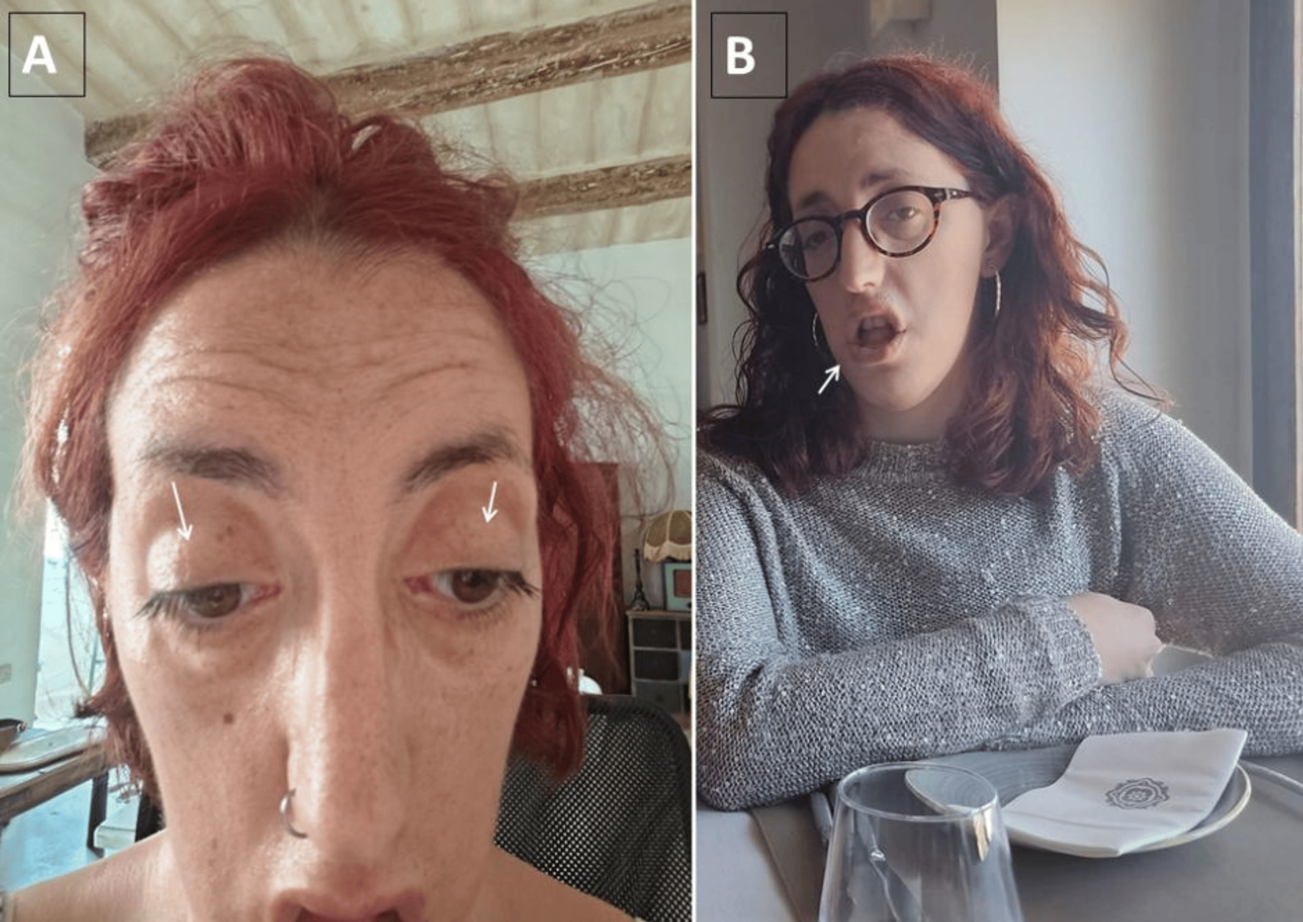
The picture of the index patient's face shows facial weakness with ptosis (A, arrows) and constantly open mouth (B, arrow) as well as dysmorphism in the form of a long, narrow face. There was also slurred speech, dysarthria, and dysphagia. The physician has received written informed consent to publish images of the patient's face.

Clinical neurological examination at the age of 36 years revealed bilateral ptosis, hypertelorism, dysarthria, open mouth, facial weakness, long narrow face with mild dysmorphism (Figure 1), mild distal weakness of the upper limbs including finger extensors, hypotonia, and mild scoliosis. She is currently taking regular pyridostigmine (360-480 mg/day) and 3,4-DAP (50-60 mg/day). Without taking these medications, the patient felt extremely tired and almost immobile.

## Discussion

The case presented is of interest to CMS because of the new homozygous variant NM_001039523:c.1396G>A in *CHRNA1*, which responded favorably to 3,4-DAP as add-on therapy to pyridostigmine. The second interesting point about this patient is that she received no treatment until the age of 17 and was still able to cope with her life, albeit with great effort. The third interesting point is that the diagnosis of CMS was made genetically with a significant delay of 29 years, which carries a certain risk of receiving medication during this period that could potentially endanger her life. Fortunately, during the period in which the genetic diagnosis was not yet known, the patient did not receive any medication that could have aggravated the clinical manifestations of CMS.

There are few reports of patients with CMS due to *CHRNA1* variants [8-15]. These patients presented phenotypically with intellectual disability, developmental delay, ocular, facial, axial, and limb muscle weakness, hypotonia, dysarthria, nasal voice, and dysphagia (Table 1). A repeatedly reported phenotypic feature of *CHRNA1* mutations that did not occur in the index patient is fatal multiple pterygium syndrome [16]. Other patients also have arthrogryposis [4]. Whether or not PCOS was a phenotypic manifestation of the *CHRNA1* mutation remains speculative. However, as PCOS has not been reported in association with a *CHRNA1* mutation or any other type of CMS, a causal relationship between CMS and PCOS is unlikely. Women carrying *CHRNA1* mutations are at least known not to worsen their phenotype during pregnancy and not to require additional or new treatment, suggesting that they can become pregnant and that pregnancy has no negative consequences for them [11].

**Table 1:** Patients with slow-channel CMS due to variants in CHRNA1 reported until September 2024 CMS: congenital myasthenic syndrome, NA: not accessible, NR: not reported

Age	Sex	Mutation	Phenotype	Treatment	Reference
NA	NA	c.806T>G	Fatigue	NA	[8]
63	Female	c.866G>T	Fluctuating, mildly progressive	Fluoxetine, non-invasive nocturnal ventilation	[9]
N=3	NR	NR	NR	NR	[10]
34	Female	c.761C>T	Ptosis, ocular, bulbar, axial, and limb weakness	None	[11]
N=5	Female (4)	c.518G>C	Ptosis, diffuse weakness, nasal voice, axial weakness	Fluoxetine	[12]
16	Female	NR	Fatigue, generalized weakness	NR	[13]
N=11	NR	c.257G>A	Facial weakness, distal weakness hypotonia, feeding difficulties, breathing difficulty	NR	[5]
N=2	NR	c.257G>A, c.119G>A	Facial weakness, distal weakness, hypotonia, feeding difficulties breathing difficulty	NR	[5]
28	Female	c.592G>A	Proximal weakness, delayed motor milestones	NR	[14]
NR	NR	G153S	NR	NR	[15]

3,4-DAP has already been shown to be beneficial in various types of CMS [2,17,18]. It has been shown to be effective in presynaptic CMS due to mutations in *SNAP25* or *SYT2*, in synaptic CMS due to mutations in *LAMA5* or *COL13A1*, in postsynaptic CMS due to mutations in *CHRND*, *CHRNE*, *FCCMS*, *MUSK*, *MYO9A*, *RAPSN*, and *SLC25A1*, or in glycosylation defects due to mutations in *GMPPB* or *DPAGT1* [2,17,18]. CMS due to *CHRNA1* variants has been rarely reported, especially for fast-channel syndrome due to *CHRNA1* [7]. First-line therapies for slow-channel syndrome due to *CHRNA1* mutations include pyridostigmine, fluoxetine, and quinidine, depending on the literature [7]. In contrast, *CHRNA1*-related slow-channel CMS is less responsive to 3,4-DAP [7]. However, most patients with *CHRNA1*-related CMS respond to pyridostigmine [4]. 3,4-DAP is only considered a second-line treatment [7]. In the few patients with *CHRNA1* who received 3,4-DAP, it was partially effective either as monotherapy or as add-on therapy [10]. In the index patient, the 3,4-DAP dosage had to be individually adapted to the patient's needs to achieve an optimal therapeutic effect. However, pyridostigmine remains the drug of first choice for *CHRNA1* carriers [7].

## Conclusions

In summary, this case shows that postsynaptic CMS with slow channels due to the new variant NM_001039523:c.1396G>A in *CHRNA1* responds well not only to pyridostigmine but also to the additional administration of 3,4-DAP. The study also shows that patients with *CHRNA1*-related CMS can live for years without treatment, especially at a young age, albeit with reduced quality of life. Patients with CMS should be diagnosed promptly to avoid exposing them to the risk of receiving medications that could potentially worsen their phenotype.
